# High‐Performance Direct Methanol Fuel Cells with Precious‐Metal‐Free Cathode

**DOI:** 10.1002/advs.201600140

**Published:** 2016-06-14

**Authors:** Qing Li, Tanyuan Wang, Dana Havas, Hanguang Zhang, Ping Xu, Jiantao Han, Jaephil Cho, Gang Wu

**Affiliations:** ^1^ State Key Laboratory of Material Processing and Die & Mould TechnologySchool of Materials Science and EngineeringHuazhong University of Science and TechnologyWuhan430074P. R. China; ^2^ Department of Chemical and Biological EngineeringUniversity at BuffaloThe State University of New YorkBuffaloNY14260USA; ^3^ School of Chemistry and Chemical EngineeringHarbin Institute of TechnologyHarbin150001P.R. China; ^4^ Department of Energy Engineering and School of Energy and Chemical EngineeringUlsan National Institute of Science and Technology (UNIST)Ulsan689‐798Republic of Korea

**Keywords:** direct methanol fuel cells, electrocatalysis, graphene, nonprecious metal catalysts, oxygen reduction

## Abstract

Direct methanol fuel cells (DMFCs) hold great promise for applications ranging from portable power for electronics to transportation. However, apart from the high costs, current Pt‐based cathodes in DMFCs suffer significantly from performance loss due to severe methanol crossover from anode to cathode. The migrated methanol in cathodes tends to contaminate Pt active sites through yielding a mixed potential region resulting from oxygen reduction reaction and methanol oxidation reaction. Therefore, highly methanol‐tolerant cathodes must be developed before DMFC technologies become viable. The newly developed reduced graphene oxide (rGO)‐based Fe‐N‐C cathode exhibits high methanol tolerance and exceeds the performance of current Pt cathodes, as evidenced by both rotating disk electrode and DMFC tests. While the morphology of 2D rGO is largely preserved, the resulting Fe‐N‐rGO catalyst provides a more unique porous structure. DMFC tests with various methanol concentrations are systematically studied using the best performing Fe‐N‐rGO catalyst. At feed concentrations greater than 2.0 m, the obtained DMFC performance from the Fe‐N‐rGO cathode is found to start exceeding that of a Pt/C cathode. This work will open a new avenue to use nonprecious metal cathode for advanced DMFC technologies with increased performance and at significantly reduced cost.

## 1 Introduction

Proton exchange membrane fuel cell (PEMFC), employing Nafion as solid polymer electrolyte, possesses many advantages including high‐energy conversion efficiency, quick start‐up, low working temperature, compactness, and no corrosion problems during the operation. Compared to other PEMFC technologies, direct methanol fuel cells (DMFCs) show the greatest promise as portable power sources due to high energy density of methanol and bypassing the handicap of storing hydrogen as fuel.^1^, ^2^, ^3^ However, their performance is limited by various factors, especially the crossover of methanol from the anode to the cathode side of the cell.^4^ Although the use of high methanol feed concentrations could be considered advantageous for DMFCs with increased energy density, freeze tolerance, ability to respond to dynamic loads, and higher limiting current densities,^5^ the crossover limits the methanol concentrations below 2.0 m in practical DMFC applications due to significant performance loss of Pt cathode. Because methanol crossover scales with methanol concentration, the increased crossover results in lower cell performance and decreased fuel efficiency, when using traditional Pt catalyst cathodes in DMFCs. From the electrochemical point of view, the degradation of Pt cathodes is due to a mixed potential region resulting from oxygen reduction reaction (ORR) and methanol oxidation reaction due to the inherent activity of Pt for both reactions. Therefore, highly methanol‐tolerant cathodes must be developed before DMFC technologies can become viable. Due to the intrinsic nature of methanol tolerance in non‐precious metal catalysts (NPMCs), the development of DMFCs provides a new opportunity for highly active NPMCs to replace expensive Pt‐based catalysts for the ORR at the cathode.

Apart from methanol contamination issue, the high cost of Pt cathodes in DMFCs also represents the most formidable challenge, preventing its much needed commercialization. In a search for high‐performance alternative NPMCs to Pt cathode over the last decade, significant progress has been made from new catalyst synthesis.^6^, ^7^, ^8^, ^9^, ^10^ Among the studied formulations, iron‐nitrogen‐carbon (Fe‐N‐C) catalysts are the most promising in terms of their activity and stability in more challenging acidic electrolytes.^11^, ^12^, ^13^, ^14^, ^15^ To date, even though substantial progress has been achieved in improving the performance of such‐synthesized Fe‐N‐C catalysts,^16^, ^17^, ^18^ their current activity in H_2_‐air fuel cell is still not comparable to Pt cathode.^19^ Practical applications of NPMCs in H_2_ fuel cells still have a long way to go. However, due to the unique intrinsic tolerance of NPMCs to methanol, they would provide a great opportunity to be used in DMFCs and achieve sufficient performance, capable of replacing current Pt cathodes.

Hence, the motivation of this work is to study the feasibility of using highly active non‐precious metal catalyst for DMFC applications. In addition, from the catalyst synthesis point of view, we have discovered a new method to prepare highly porous graphene catalyst for the ORR cathode in DMFCs. Traditionally, carbon blacks including Ketjenblack (KJ) and BlackPearl have been extensively studied during the preparation of Fe‐N‐C catalysts due to their high surface areas (above 700 m^2^ g^–1^) and porosity. Alternatively, since the discovery of graphene, interest in graphene oxide (GO) or reduced GO (rGO) as a novel graphene‐support for electrocatalysts in fuel cell applications has grown rapidly. However, due to the relatively low surface area of rGO around 200–300 m^2^ g^–1^,^20^ the rGO‐based Fe‐N‐C catalysts did not exhibit superior activity, yet, in relation to carbon‐black‐based ones, especially in more challenging acidic electrolytes.^14^ Therefore, development of rGO‐based catalysts with high surface areas still remains a grand challenge. In this work, an rGO‐based Fe‐N‐C catalyst that processes unique highly porous morphology is derived from a simple nitrogen precursor (i.e., melamine), iron chloride, and microwave treated rGO. The newly synthesized Fe‐N‐rGO catalyst yielded a high ORR activity in acid comparable to other state‐of‐the‐art NPMCs. It also exhibited superior methanol tolerance in rotating disk electrode (RDE) relative to commercial Pt/C catalysts. Importantly, the newly synthesized Fe‐N‐rGO catalyst was further employed to fabricate a cathode and implemented into a membrane electrode assembly (MEA) for DMFC tests under various realistic DMFC operational conditions. The performance of the Fe‐N‐rGO cathode exceeded the performance of Pt/C cathode when the methanol feed concentration was higher than 2.0 m, demonstrating viable possibility of using earth‐abundant catalysts for DMFC technologies.

## 2 Results and Discussion

**Figure 1:**
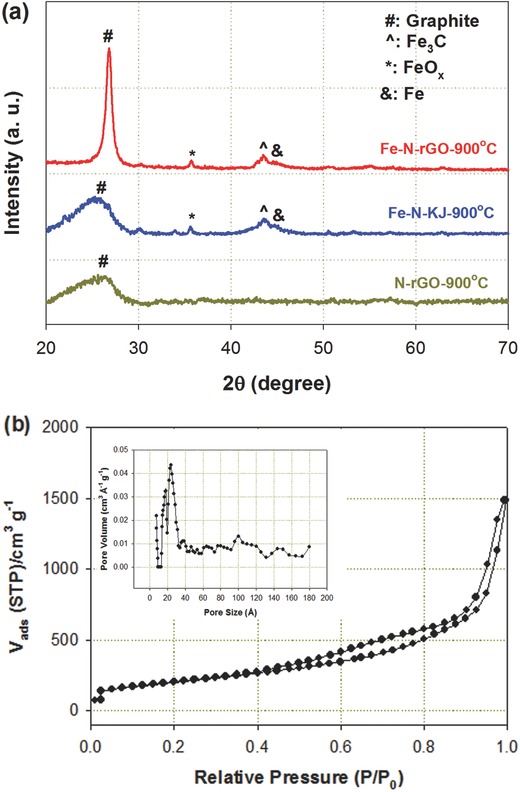
a) XRD patterns of various catalysts, and b) nitrogen adsorption–desorption isotherm and pore size distribution curve (inset) of Fe‐N‐rGO‐900 °C catalyst.

Considering the Fe‐N‐C catalyst synthesis, structural similarities between the aromatic nitrogen precursors and graphite has attracted much attention regarding the synthesis of M‐N‐C catalysts.^21^ To increase nitrogen content in the graphitized carbon structure, melamine, a trimer of cyanamide, with a 1,3,5‐triazine skeleton containing 66 wt% nitrogen, has shown to be a promising nitrogen precursor.^22^ More importantly, melamine additives were found to be able to efficiently exfoliate graphite into high‐quality graphene sheets due to the melamine‐induced hydrophilic force from the basal plane.^23^ Thus, the use of melamine for the rGO‐based catalyst synthesis facilitates in situ protection of the graphene flake agglomeration, leading to good dispersion among rGO, melamine, and iron precursors. The synthesis of the Fe‐N‐C catalysts, in this work, began with adding a specific amount of melamine, ammonium peroxydisulfate (APS), FeCl_3_, and rGO into hydrochloric solution while stirring; the resulting powders, after drying, were annealed at 350 °C and then at 800–1000 °C. In principle, melamine would melt and therefore spread with the coordinated metal ions over the rGO surface at temperatures higher than 350 °C before its decomposition. Some melamine molecules may be adsorbed on the graphene surface through π–π interaction, allowing uniform and high‐density *N*‐doping of the graphene sheets. In addition, the nitrogen‐containing polymer (e.g., polymeric melem^24^), evolved from melamine during pyrolysis, was decomposed along with the simultaneous release of a large amount of carbon nitride gases (e.g., C_2_N_2_^+^, C_3_N_2_^+^, and C_3_N_3_^+^).^25^ These gases evolve into the *N*‐doped graphene structures and coordinate with Fe to generate Fe‐N*_x_* active sites. **Figure** **1**a presents the X‐ray diffraction (XRD) pattern of the Fe‐N‐rGO, Fe‐N‐KJ, and iron‐free N‐rGO catalysts heat treated at 900 °C. As for the Fe‐N‐rGO‐900°C catalyst, the diffraction peak at 26.5° corresponds to the (002) planes of graphitic carbon, while the peaks at 35.6° and 62.9° indicate the presence of Fe_3_O_4_ species (JCPDS, No. 89‐3854). Importantly, the peaks at 43.7° and 44.8° indicate the presence of large amounts of Fe_3_C (JCPDS, No. 89‐2867) and α‐Fe species (JCPDS, No. 87‐0722). The XRD pattern of Fe‐N‐KJ‐900 °C is comparable with that of Fe‐N‐rGO‐900°C. In addition, no significant characteristic peaks of Fe species can be observed in the XRD pattern of the N‐rGO‐900°C catalyst. Table S1 (Supporting Information) summarizes the effect of heating temperature on elemental composition and BET surface areas of the Fe‐N‐rGO catalysts. Compared to other temperatures, the 900 °C treatment leads to the highest BET surface area of 732 m^2^ g^−1^, which is well correlated with the highest ORR activity (vide infra). It should be noted that the highest BET surface area resulting from 900 °C is due to the in situ formed iron sulfide (FeS) during pyrolysis from FeCl_3_ and ammonium persulfate, which acts as an effective sacrificial pore‐forming agent and can be efficiently leached out during the acid treatment. From Table S1 (Supporting Information), the Fe and S contents of Fe‐N‐rGO‐900 °C catalyst are the lowest compared to that of Fe‐N‐rGO pyrolyzed at 800 and 1000 °C, which means the FeS in Fe‐N‐rGO‐900 °C catalyst can somehow more effectively leach away, thereby leading to the highest BET surface area among three NPMCs. In addition, the high surface area of Fe‐N‐rGO‐900 °C relative to that of microwave‐treated rGO (≈450 m^2^ g^−1^) and Fe‐free N‐rGO‐900 °C (≈229 m^2^ g^−1^) catalysts can also be primarily attributed to the efficient removal of in situ generated FeS species,^21^, ^26^ as evidenced by the absence of FeS features in the XRD pattern of the resulting catalyst after acidic leaching treatment. In particular, the sample, pyrolyzed at 900 °C, produced a Type I/IV hybrid isotherm indicating a micro/mesoporous structure (Figure 1b). The micro/mesoporous structure is mainly attributed to micropores with diameter ranging from 1.5 to 2.5 nm in the Fe‐N‐rGO catalyst (Figure 1b inset).

**Figure 2:**
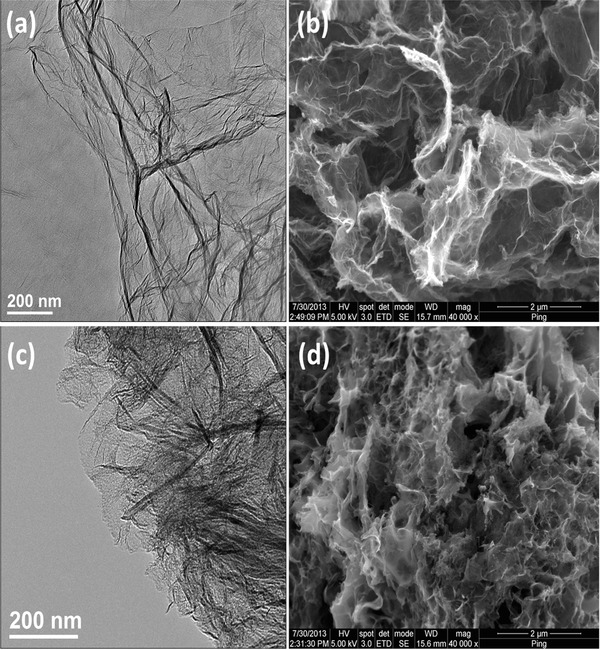
TEM and SEM images for a,b) N‐rGO and c,d) Fe‐N‐rGO‐900 °C.

**Figure 3:**
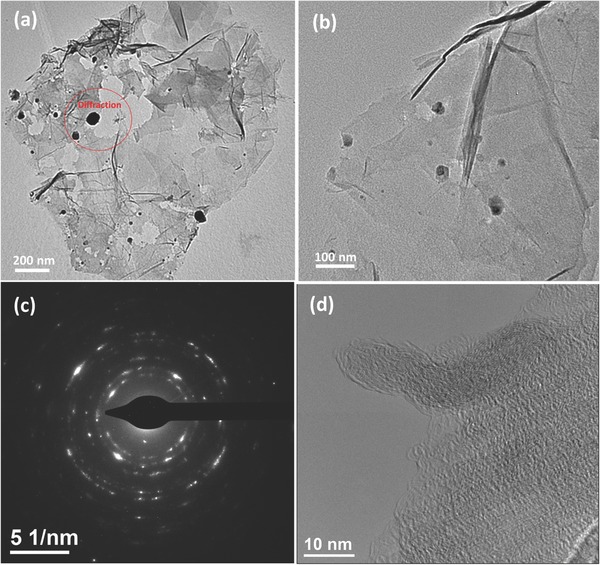
TEM images and electron diffraction for highly porous Fe‐N‐rGO‐900 °C catalysts.

The overall morphology of N‐rGO and Fe‐N‐rGO determined by transmission electron microscopy (TEM) and scanning electron microscopy (SEM) was compared in **Figure** **2**. Compared to the heat treated rGO (Figure S1, Supporting Information) and N‐rGO samples (Figure 2a,b), the graphene‐like structure is primarily attributed to rGO, rather than the pyrolysis of melamine. This was also determined from the observation of the control sample derived from melamine, iron, and KJ carbon (Fe‐N‐KJ‐900 °C), where no graphene structures are observed except for graphitized carbon (Figure S2, Supporting Information). It should be noted that much more porous morphology was observed with Fe‐N‐rGO sample, relative to iron‐free N‐rGO. Interestingly, in the Fe‐N‐rGO catalyst, significant “holes” (100–200 nm) are observed in some graphene sheets based on TEM images (**Figure** **3** **;** Figure S3, Supporting Information).^27^ Therefore, the highly porous structures likely result from the addition of FeCl_3_ due to its strong oxidative capability especially during the high‐temperature treatment and subsequent acid leaching. Previous work has demonstrated that pores of 100–200 nm, in the graphene sheets, can be created via processing with a strong oxidant such as ozone and iron (III) chloride.^28^ The resulting porous graphene could efficiently reduce the mass diffusion resistance and increase the surface area compared to pristine graphene sheets, greatly enhancing its electrocatalytic activity.^27^, ^29^ Therefore, the high BET surface area up to 732 m^2^ g^−1^ of the new Fe‐N‐rGO NPMC prepared in this work may be partially attributed to such “holes” in graphene sheets. Traditional rGO‐based NPMCs usually suffer from low surface area due to absence of microporous structures, thereby leading to relatively low ORR activity relative to the carbon‐black derived one.^14^ However, in this work, using FeCl_3_ and melamine as additives, the highly porous rGO catalyst featured with enhanced surface areas and dominant mesoporous structures holds great promise to facilitate the ORR. Figure 3 also exhibits selected area electron diffraction of metal particles observed on the Fe‐N‐rGO catalyst, suggesting different crystal structures of Fe/Fe_3_C that are by‐products during the high temperature treatment and likely inactive for the ORR. Meanwhile, the high‐resolution TEM (HRTEM) images shows evidence of disordered edge structures for the rGO‐based catalysts, which were believed to be active sites for O_2_ adsorption.^30^

**Figure 4:**
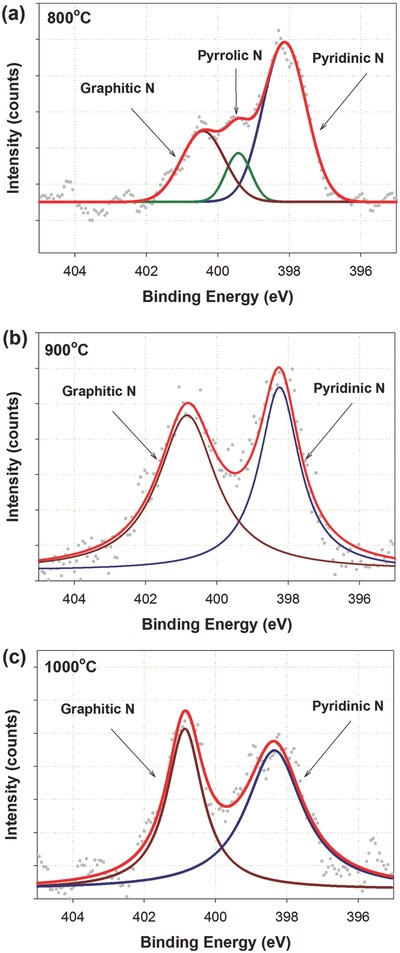
N 1s XPS spectra of Fe‐N‐rGO catalysts heattreated at a) 800, b) 900, and c) 1000 °C.

During the catalyst synthesis, we first studied the heating temperature for the Fe‐N‐rGO catalysts in terms of resulting nitrogen doping and ORR activity. Table S1 (Supporting Information) indicates a total nitrogen content that consistently decreases from 4.07 to 2.98 at% with increasing heating temperature from 800 to1000 °C, which does not lead to a corresponding drop in the ORR activity (vide infra). These data suggest that ORR activity on this type of NPMC is not dependent on the total doped nitrogen atoms, but more likely on how the nitrogen is doped into the carbon structures and coordinated with metal to form Fe‐N*_x_* active site. The high‐resolution N 1s X‐ray photoelectron spectroscopy (XPS) spectra of Fe‐N‐rGO catalysts heat treated at 800–1000 °C show two dominant nitrogen peaks at ≈400.8 and 398.3 eV (**Figure** **4**), which are assigned to graphitic and pyridinic nitrogen, respectively.^31^, ^32^ In addition, the pyrrolic form of nitrogen (399.5 eV) observed at the N 1s spectrum of Fe‐N‐rGO‐800 °C is assigned to nitrogen atoms in a pentagon structure.^11^, ^13^, ^31^, ^33^ Pyrrolic nitrogen has been shown to decompose at temperatures above 800 °C to either pyridinic or graphitic nitrogen.^31^, ^33^, ^34^, ^35^ The successful replacement of carbon atoms inside of the graphitic lattice (graphitic N) with nitrogen is often connected with ORR active sites,^36^ however, incorporation of nitrogen at the center of graphitic sheets has only recently been correlated with enhanced onset potential during the ORR due to significant changes of electron distribution on carbon planes.^37^, ^38^ Interestingly, the ratio of graphitic to pyridinic nitrogen goes up as the heating temperature is increased, while the ORR activity reaches a maximum at 900 °C. This data indicates that not only is the presence of pyridinic and graphitic nitrogens necessary for efficient ORR activity, but also that the ratio of nitrogen doped structures can significantly impact catalyst activity.

**Figure 5:**
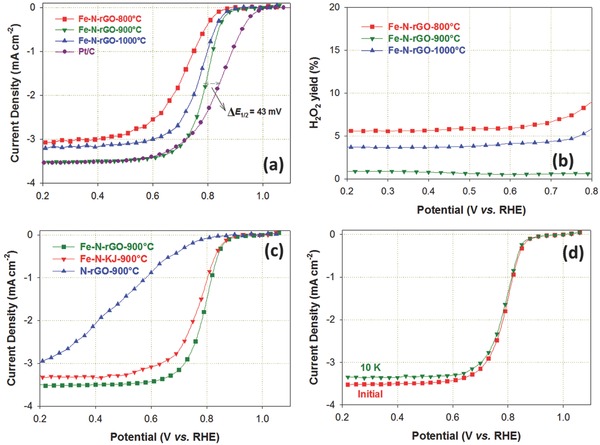
a) ORR polarization plots of Fe‐N‐rGO‐900, Fe‐N‐KJ‐900, and N‐rGO‐900 °C catalysts in 0.5 m H_2_SO_4_. Rotating speed: 900 rpm. b) ORR activity and c) H_2_O_2_ yield of Fe‐N‐rGO catalysts as a function of heating temperature. d) Durability test of the Fe‐NrGO‐900 °C catalyst by cycling in nitrogen‐gas in the potential range from 0.6 to 1.0 V.

In catalyst synthesis chemistry, the heat treatment temperature is a chief factor in inducing catalytic activity of the Fe‐N‐C type catalysts and assuring performance stability. We used an rotating ring‐disk electrode (RRDE) to study the ORR activity (**Figure** **5**a) and H_2_O_2_ yields (Figure 5b) of an Fe‐N‐rGO catalyst as a function of heat treatment temperatures ranging from 800 to 1000 °C. RRDE studies were conducted at room temperature in 0.5 m H_2_SO_4_ electrolyte. Activity, measured by the ORR onset and half‐wave potentials (*E*_1/2_) in the RDE polarization plots, increased upon raising the heat treatment temperature to 900 °C and then dropped for catalysts synthesized at even higher temperatures of 1000 °C. The activity gap between the state‐of‐the‐art Pt/C (E‐TEK) and the Fe‐N‐rGO‐900°C catalyst, as reflected by a difference of half‐wave potential (Δ*E*_1/2_) in RDE testing, has been substantially reduced to ≈60 mV (0.85 vs 0.79 V). The measured ORR activity in acidic media is comparable to that of advanced NPMCs.^9^ The Fe‐N‐rGO‐900 °C catalyst also demonstrates superior ORR activity in comparison to Fe‐N‐KJ‐900 °C and N‐rGO‐900 °C catalysts (Figure 5c). Relatively poor ORR activity of metal‐free N‐rGO‐900 °C catalyst was observed as expected, due to the lack of Fe‐N*_x_*/C active sites. Lower activity of the Fe‐N‐KJ‐900 °C catalyst, in comparison to Fe‐N‐rGO‐900°C catalyst, can be attributed to the smaller BET surface area (579 m^2^ g^−1^ vs 732 m^2^ g^−1^). Fe‐N‐rGO‐900 °C catalyst also has the lowest H_2_O_2_ field (≈1%) relative to the two Fe‐N‐rGO catalysts at 800 and 1000 °C, which is in good agreement with their ORR activity, signaling virtually complete reduction of O_2_ to H_2_O in a four‐electron process. The Tafel slope (*b*) was calculated from kinetic current density (*j*_k_) to evaluate the ORR mechanism on these catalysts. According to the Koutecky–Levich equation (Equation (1)), *j*_k_ is derived from the steady‐state (*j*) and diffusion‐limiting current density (*j*_d_)^39^, ^40^

(1) $$j_{k}=j\times j_{d}/(j_{d}-j)$$

Figure S4 (Supporting Information) shows the representative Tafel plots of ORR on Fe‐N‐rGO catalysts heat treated at different temperatures. Theoretically, a Tafel slope of 120 mV dec^−1^ represents the rate‐determining step associated with the first‐electron transfer, while a Tafel slope of 60 mV dec^−1^ represents the migration rate of adsorbed oxygen intermediates with a Temkin isotherm.^41^ In this work, Tafel slopes measured with the Fe‐N‐rGO catalysts are close to 67 mV dec^−1^, suggesting that the intermediate migration in ORR on these catalysts may be the rate determining step. On the other hand, high stability of the Fe‐N‐rGO‐900 °C catalyst is demonstrated in potential cycling tests (Figure 5d). The cycling was carried out within a potential range of 0.6 to 1.0 V in nitrogen‐saturated 0.5 m H_2_SO_4_ at a scan rate of 50 mV s^−1^.^9^ No significant activity loss was observed in the ORR kinetic region of Fe‐N‐rGO‐900 °C catalyst even after 10 000 cycles, attesting to the high durability of the developed catalysts in acidic electrolyte.

**Figure 6:**
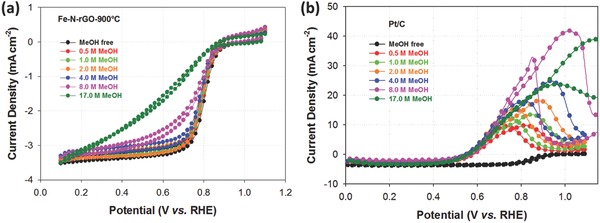
ORR activity measured with a) Fe‐N‐rGO‐900 °C and b) Pt/C (20 μgPt cm^–2^) catalysts as a function of methanol concentration.

The effect of methanol contamination on overall ORR activity for both Fe‐N‐rGO‐900 °C and traditional Pt/C catalysts was studied in an O_2_‐saturated 0.5 m sulfuric acid solution as a function of methanol concentration from 0.5 to 17.0 m (**Figure** **6**). The Fe‐N‐rGO‐900°C catalyst (Figure 6a) shows that ORR activity in the kinetic range of the polarization curves is nearly independent of the addition of methanol. Although the *E*_1/2_ of ORR measured with Fe‐N‐rGO‐900 °C catalyst shifts in the negative direction by only ≈200 mV after adding 17.0 m methanol, it is attributed to the significant reduction of O_2_ concentration in the electrolyte. These results indicate superior methanol tolerance of the Fe‐N‐rGO catalyst. By contrast, the ORR characteristics on the Pt/C catalyst are thoroughly overwhelmed even in the presence of 0.5 m methanol (Figure 6b), indicative of extremely poor methanol tolerance. Each of the ORR activity curves, in the presence of methanol, reveals typical features of methanol oxidation on Pt catalysts with one oxidative peak at positive scan and another oxidative peak at negative scan.^42^, ^43^

**Figure 7:**
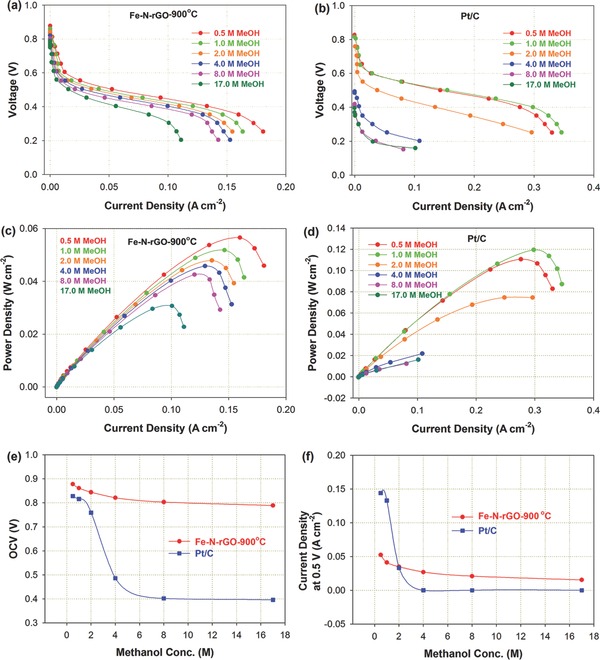
a,b) DMFC cell voltage and c,d) power density versus current density measured with (a,c) Fe‐N‐rGO‐900 °C and b,d) Pt/C catalysts as a function of methanol feed concentration. e) OCV and f) current density at 0.5 V of both catalysts as a function of methanol feed concentration. Anode: 2.7 mgPt cm^–2^ PtRu/C, 1.8 mL min^–1^ MeOH solution; cathode: 4 mg cm^–2^ Fe‐N‐rGO‐900 °C or 2.0 mgPt cm^–2^ Pt/C, 500 sccm air; membrane: 2 × Nafion 212; cell: 75 °C.

DMFC voltage and power density as a function of current density obtained with the Fe‐N‐rGO‐900 °C catalyst, and the standard Pt/C reference catalyst, at the cathode are shown in **Figure** **7**a,c and 7b,d, respectively. Open circuit voltage (OCV) values measured with Fe‐N‐rGO and Pt/C catalysts, as a function of methanol feed concentration, are compared in Figure 7e. In general, methanol crossover will substantially decrease the OCV of a DMFC based on a Pt cathode, due to the formation of mixed potentials resulting from the high methanol oxidation activity of Pt catalysts.^44^ Apparently, the OCV measured with Fe‐N‐rGO‐900 °C catalyst is higher than that of Pt/C even at the lowest methanol feed concentration of 0.5 m (0.875 V vs 0.826 V). This demonstrates an outstanding methanol tolerance of the developed NPMC. Further increasing the methanol concentration significantly enlarged the gap between the OCV values recorded on these two catalysts. In addition, the current densities obtained at 0.5 V, with the two studied catalysts, are listed in Figure 7f. At 0.5 m methanol feed concentration, the current density at 0.4 V obtained with the Pt cathode is ca. two times higher than that recorded with the Fe‐N‐rGO cathode (0.275 vs 0.135 mA cm^−2^), revealing the still‐existing performance gap between the non‐precious cathode catalyst and Pt catalyst for the DMFC cathode. With increasing the methanol concentration to 2.0 m, the DMFC performance obtained with Fe‐N‐rGO catalyst is comparable with that of Pt/C catalyst (0.115 vs 0.120 mA cm^−2^ at 0.4 V). Furthermore, when the methanol concentration exceeding 2.0 m, the fuel cell with Pt/C cathode cannot deliver any current at 0.5 V, again, indicating the serious limitation of Pt‐based cathodes at high methanol concentrations. Therefore, the implementation of an NPMC at the cathode of a DMFC provides the great opportunity for allowing high methanol concentration in more practical DMFC technology, potentially benefiting energy density, freeze tolerance, ability to respond to dynamic loads, and limiting current densities.

## 3 Conclusion

The performance of DMFCs using Pt/C cathodes is significantly reduced due to the severe methanol crossover from anode to cathode sides, especially with increased methanol feed concentrations. In this study, a heat‐treated Fe‐N‐rGO NPMC, derived from aromatic nitrogen precursors (i.e., melamine), iron, and rGO, was developed for catalyzing ORR at the DMFC cathodes. The results demonstrate that the Fe‐N‐rGO catalysts are capable of tolerating highly‐concentrated methanol, up to 4.0 m, without significant performance loss. This NPMC also exhibits superior ORR activity and cycle stability in acidic electrolyte. The heating temperature of 900 °C was found to generate the best ORR activity in the final catalysts relative to other pyrolysis temperatures. The optimal temperature is associated with the highest BET surface area of 732 m^2^ g^−1^ and a dominant micro/mesoporous structure. Importantly, the DMFC performance measured with the best‐performing NPMC (Fe‐N‐rGO‐900 °C), at 2.0 m methanol feed concentration, starts to exceed that of the currently best reported Pt/C catalyst and achieves the specific goal of the DMFC cathode catalyst research with current density > 0.1 A cm^−2^ at 0.4 V. Due to the superior methanol tolerance and high ORR activity of the developed Fe‐N‐rGO catalyst, the DMFC performance using the NMPC cathode is able to outperform that of Pt/C cathode, paving the way for employing increased methanol concentration as well as significnatly reduce the cost for advanced DMFC technologies.

## 4 Experimental Section

*Material Synthesis*: In a typical approach to preparing the Fe‐N‐rGO catalysts, 2.0 g melamine was dispersed with 0.4 g rGO in a 1.0 m HCl solution. The rGO was reduced from GO through a microwave treatment. GO aqueous solution was prepared using the Hummers′ method by using several strong oxidants, such as potassium permanganate, sodium nitrate, and sulfuric acid to treat natural graphite powder.^45^ The pore agent (APS) and transition metal precursors (FeCl_3_) were then added. After constant stirring for 24 h, the solvent was evaporated at 60 °C. The remaining catalyst powders were first heat treated at 350 °C for 0.5 h and then pyrolyzed at elevated temperatures ranging from 800 to 1000 °C for 1 h, both in an N_2_ atmosphere. The heat‐treated sample was then preleached in 0.5 m H_2_SO_4_ at 80 °C for 8 h to remove unstable and inactive species from the catalyst followed by thorough washing with deionized water. Finally, the catalyst was heat treated again at 800 °C 800–1000 °C in an N_2_ atmosphere for 3 h. The final catalysts were labelled as Fe‐N‐rGO‐800°C, Fe‐N‐rGO‐900 °C, and Fe‐N‐rGO‐1000 °C, respectively. The control sample derived from annealing melamine, iron, and KJ black at 900 °C was denoted as Fe‐N‐KJ‐900 °C. The one derived from annealing melamine and rGO at 900 °C was denoted as N‐rGO‐900 °C.

*Physical Characterization*: Catalyst morphology was characterized by SEM using a FEI Quanta 400 ESEM. HRTEM images were taken with a JEOL 3000F TEM. Surface area of the carbon‐based catalysts was measured using the Brunauer–Emmett–Teller method on an Autosorb‐IQ/MP‐XR instrument with nitrogen adsorption at 77 K. Pore‐size distribution was determined from the adsorption isotherm using density functional theory with slit pore geometry (Quantachrome analysis software). The crystallinity of samples was determined by XRD using a Bruker AXS D8 Avance diffractometer with Cu KR radiation. XPS was performed on an ESCA 210 and MICROLAB 310D spectrometer using a Mg KR source.

*Electrochemical Characterization*: ORR activity and selectivity of catalyst samples were electrochemically evaluated on RRDE. The electrochemical tests were carried out on a CHI Electrochemical Station (Model 750b) in a conventional three‐electrode cell at room temperature. A graphite rod and an Hg/HgSO_4_ electrode in 0.5 m H_2_SO_4_ were used as the counter and reference electrodes, respectively. 0.5 m H_2_SO_4_ was used as the electrolyte to test ORR activity. The catalyst loading was controlled at 0.6 mg cm^−2^. Pt reference data were recorded with a 20 wt% E‐TEK Pt/C catalyst at a loading of 20 μg_Pt_ cm^−2^. ORR steady‐state RDE polarization plots were recorded in O_2_‐saturated electrolytes using a potential step of 0.03 V and wait‐period of 30 s between two subsequent potentials. The disk rotation rate was 900 rpm. In RRDE experiments, the ring potential was set to 1.2 V. Before performing the experiments, the Pt catalyst in the ring was activated by potential cycling in 0.5 m H_2_SO_4_ from 0 to 1.4 V at a scan rate of 50 mV s^−1^ for 10 min. Potential cycling was carried out within a potential range of 0.6 to 1.0 V in nitrogen gas at a scan rate of 50 mV s^−1^.

*MEA Preparation*: MEAs were fabricated using 2 × Nafion 212 membranes in an acid form with catalyst inks. 75 wt% Pt_50_Ru_50_/C (Johnson Matthey) was used as the anode catalyst. Pt/C (Johnson Matthey) and as‐synthesized Fe‐N‐rGO were used as the cathode catalysts for Pt‐based and NPMC‐based MEAs, respectively. The inks were prepared by ultrasonically mixing appropriate amounts of catalyst powders with deionized water (Millipore, 18 MΩ cm) and 5% Nafion suspension (Ion Power, Inc.) for 90 s. Subsequently, the inks were brush‐painted onto the membrane at 75 °C and dried for 30 min. The anode catalyst loading was 2.7 mg_Pt_ cm^−2^. The cathode catalyst loading was 2.0 mg_Pt_ cm^−2^ and 4.0 mg cm^−2^ for Pt/C and Fe‐N‐rGO catalysts, respectively. The active cell area was 5 cm^2^.

*Fuel Cell Tests*: DMFC testing was carried out in a single cell using a commercial fuel cell test system (Arbin FCTs instrument). The MEA was sandwiched between two graphite plates machined with single‐serpentine flow channels in them. The cell was operated at 75 °C, a standard operating temperature for a DMFC. Methanol solution at various concentrations (0.5, 1.0, 2.0, 4.0, 8.0, and 17.0 M) was delivered to the anode at a flow rate of 1.8 mL min^−1^ using a high‐pressure liquid chromatography pump. Humidified air was supplied to the cathode at a flow rate of 500 standard cubic centimeters per minute (sccm) at ambient pressure. To measure high frequency resistance, a sinusoidal voltage perturbation between 2 and 10 kHz (chosen to minimize capacitance) was applied to the fuel cell load. Hydrogen/air polarization plots were recorded before DMFC testing, to primarily assess the cathode performance.

## Supporting information

As a service to our authors and readers, this journal provides supporting information supplied by the authors. Such materials are peer reviewed and may be re‐organized for online delivery, but are not copy‐edited or typeset. Technical support issues arising from supporting information (other than missing files) should be addressed to the authors.

Supplementary
