# Supplementary material for: High‐Performance Direct Methanol Fuel Cells with Precious‐Metal‐Free Cathode
Source: Adv Sci (Weinh). 2016 Jun 14;3(11):1600140. doi: 10.1002/advs.201600140 (PMC5102660; doi:10.1002/advs.201600140)
Supplement: Supplementary file 1 — Supplementary [file ADVS-3-0b-s001.pdf]

Copyright WILEY-VCH Verlag GmbH & Co. KGaA, 69469 Weinheim, Germany, 2016.

Supporting Information

for *Adv. Sci.,* DOI: 10.1002/advs.201600140

High-Performance Direct Methanol Fuel Cells with Precious-Metal-Free Cathode

Qing Li, Tanyuan Wang, Dana Havas, Hanguang Zhang, Ping Xu, Jiantao Han, Jaephil Cho,* and Gang Wu**

Supporting Information

for

High-performance Direct Methanol Fuel Cells with Precious Metal-free Cathode

Qing Li[1,*], Tanyuan Wang[1], Dana Havas[2], Hanguang Zhang[2], Ping Xu[3], Jiantao Han[1], Jaephil Cho[4,*], and Gang Wu[2,*]

[1] State Key Laboratory of Material Processing and Die & Mould Technology, School of Materials Science and Engineering, Huazhong University of Science and Technology, Wuhan 430074, China

[2]Department of Chemical and Biological Engineering, University at Buffalo, The State University of New York, Buffalo, NY 14260, United States

[3]Department of Chemistry, Harbin Institute of Technology, Harbin 150001, China.

[4] Department of Energy Engineering and School of Energy and Chemical Engineering, Ulsan National Institute of Science and Technology (UNIST), Ulsan 689-798, Republic of Korea

*Corresponding authors:

E-mail addresses: qing_li@hust.edu.cn (Q. Li), jpcho@unist.ac.kr (J.P. Cho), and gangwu@buffalo.edu (G. Wu)

Table S1. Elemental composition and BET surface area of Fe-N-rGO catalysts as a function of heating temperature.

Heating Temperature (°C)	Atomic concentration (%)					BET surface area (m^2 g^{-1})
	S	N	C	Fe	O	
800	0.61	4.07	86.81	2.43	6.08	605
900	0.51	3.63	89.85	1.58	2.60	732
1000	1.18	2.98	89.55	4.3	2.0	646

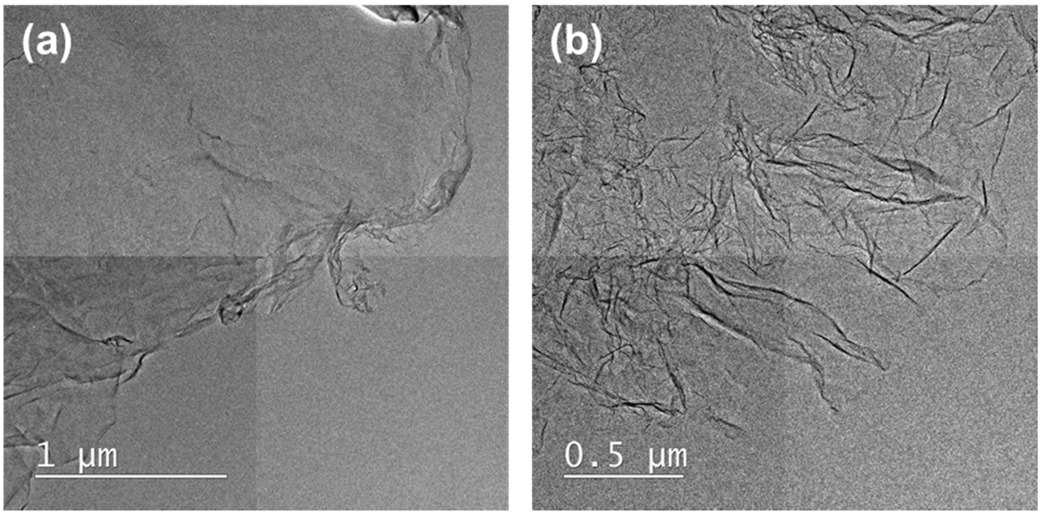

Figure S1. TEM images of rGO samples after a heat treatment at 900°C and acid leaching treatment in 0.5 M H_2SO_4 at 80 ^{o}C

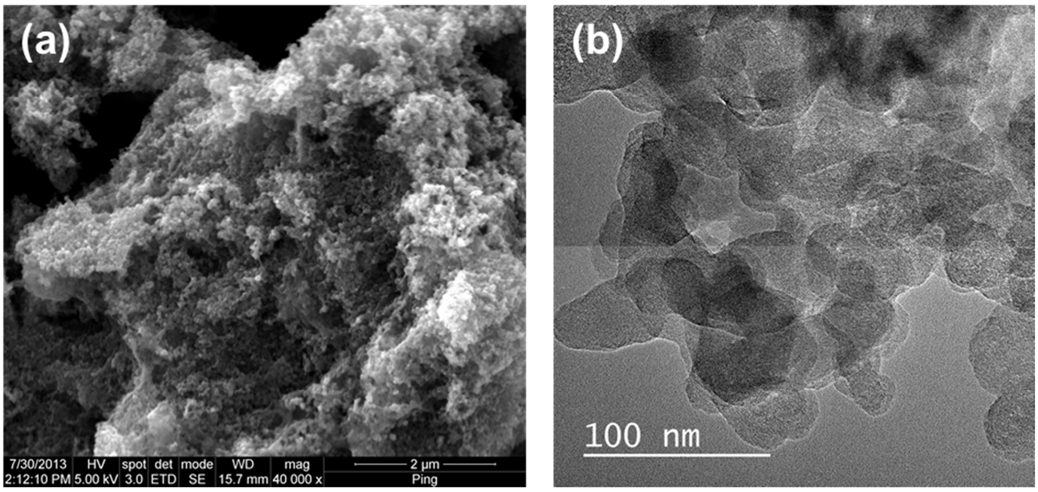

Figure S2. (a) SEM and (b) TEM images of iron-free N-KJ black catalyst heat-treated at 900°C.

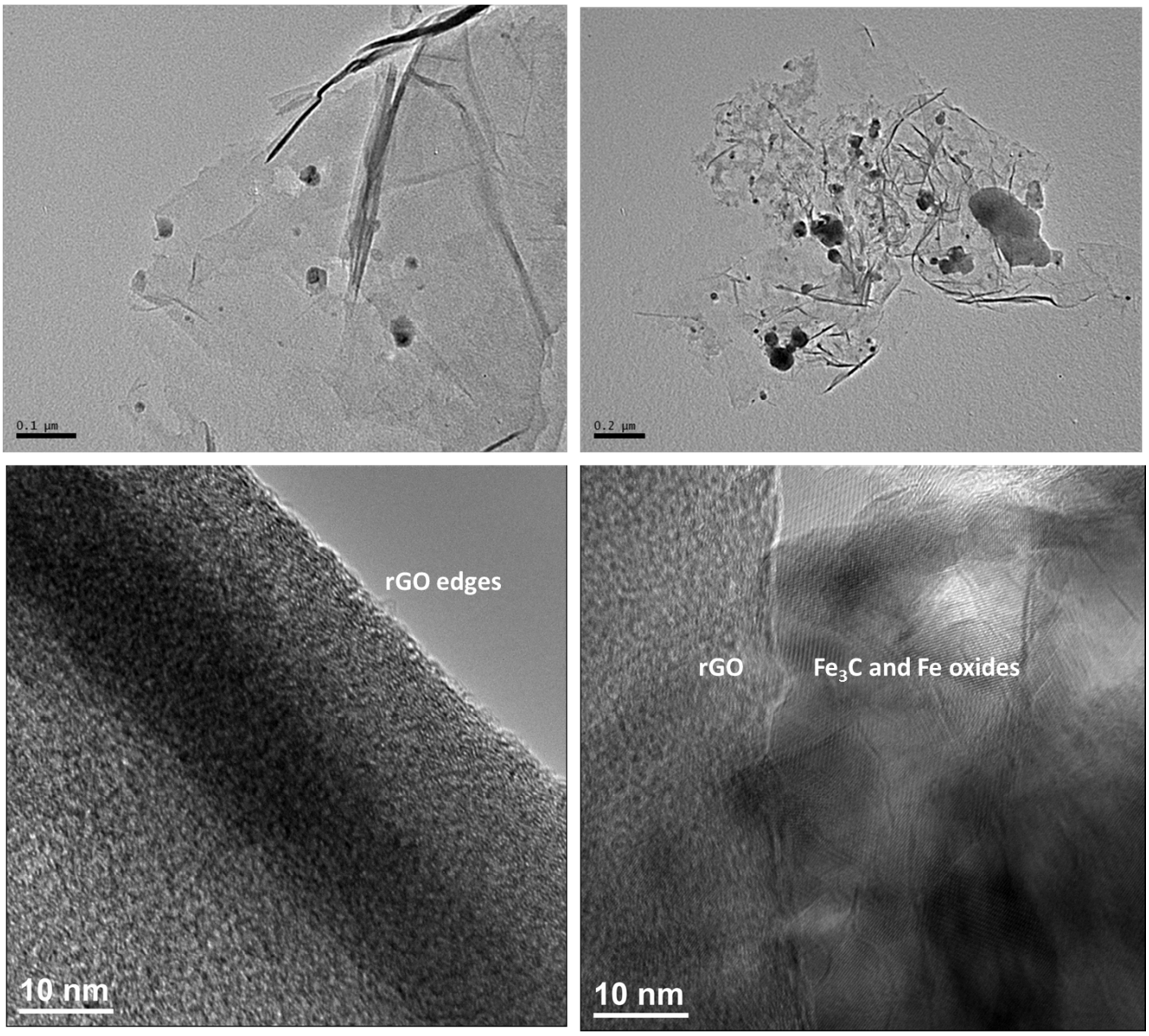

Figure S3. Additional TEM and HR-TEM images of Fe-N-rGO-900°C catalysts.

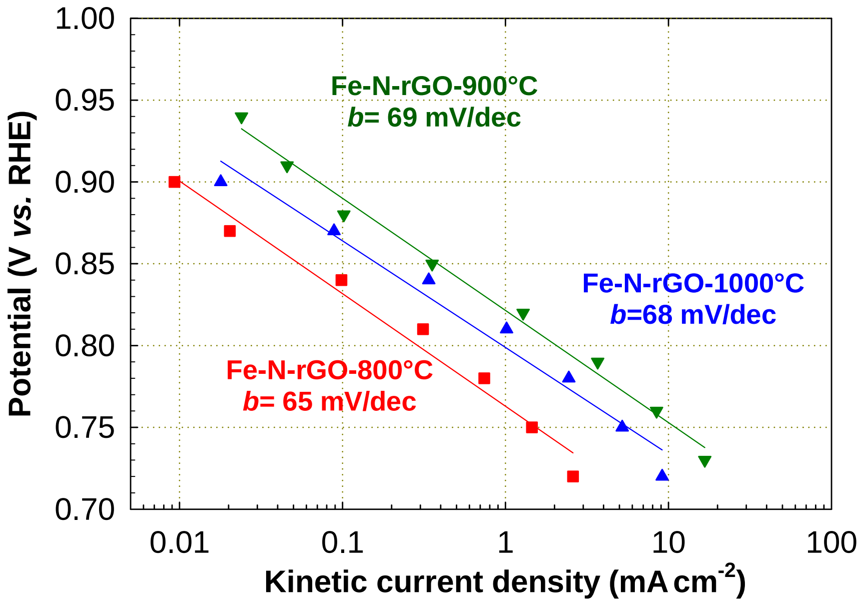

Figure S4. Tafel plots for the ORR on Fe-N-rGO catalysts heat-treated at different temperatures.
